# Surface Properties, Free Volume, and Performance for Thin-Film Composite Pervaporation Membranes Fabricated through Interfacial Polymerization Involving Different Organic Solvents

**DOI:** 10.3390/polym12102326

**Published:** 2020-10-12

**Authors:** Micah Belle Marie Yap Ang, Shu-Hsien Huang, Shi-Wei Wei, Yu-Hsuan Chiao, Ruth R. Aquino, Wei-Song Hung, Hui-An Tsai, Kueir-Rarn Lee, Juin-Yih Lai

**Affiliations:** 1 R&D Center for Membrane Technology, Department of Chemical Engineering, Chung Yuan Christian University, Taoyuan 32023, Taiwan; mbmyang@gmail.com (M.B.M.Y.A.); iceiwine@hotmail.com (S.-W.W.); ychiao@uark.edu (Y.-H.C.); wshung@mail.ntust.edu.tw (W.-S.H.); krlee@cycu.edu.tw (K.-R.L.); jylai@mail.ntust.edu.tw (J.-Y.L.); 2 Department of Chemical and Materials Engineering, National Ilan University, Yilan 26047, Taiwan; 3 Department of Chemical Engineering, University of Arkansas, Fayetteville, AR 72701, USA; 4 General Education Department, Colegio de Muntinlupa, Mayor J. Posadas Avenue, Sucat, Muntinlupa City 1770, Metro Manila, Philippines; ruthraquino@yahoo.com; 5 School of Chemical, Biological, and Materials Engineering and Sciences, Mapúa University, Manila 1002, Philippines; 6 Advanced Membrane Materials Research Center, Graduate Institute of Applied Science and Technology, National Taiwan University of Science and Technology, Taipei 10607, Taiwan; 7 Research Center for Circular Economy, Chung Yuan Christian University, Taoyuan 32023, Taiwan

**Keywords:** thin-film composite membranes, pervaporation, interfacial polymerization, polyamide, organic solvent

## Abstract

The type of organic solvents used in interfacial polymerization affects the surface property, free volume, and separation performance of the thin-film composite (TFC) polyamide membrane. In this study, TFC polyamide membrane was fabricated through interfacial polymerization between diethylenetriamine (DETA) and trimesoyl chloride (TMC). Four types of organic solvent were explored in the preparation of pervaporation membrane. These are tetralin, toluene, hexane, and isopentane. The solubility parameter distance between organic solvents and DETA follows in increasing order: tetralin (17.07 MPa^1/2^) < toluene (17.31 MPa^1/2^) < hexane (19.86 MPa^1/2^) < isopentane (20.43 MPa^1/2^). Same trend was also observed between the organic solvents and DETA. The larger the solubility parameter distance, the denser and thicker the polyamide. Consequently, field emission scanning electron microscope (FESEM) and positron annihilation spectroscopy (PAS) analysis revealed that TFC_isopentane_ had the thickest polyamide layer. It also delivered the highest pervaporation efficiency (permeation flux = 860 ± 71 g m^−2^ h^−1^; water concentration in permeate = 99.2 ± 0.8 wt%; pervaporation separation index = 959,760) at dehydration of 90 wt% aqueous ethanol solution. Furthermore, TFC_isopentane_ also exhibited a high separation efficiency in isopropanol and tert-butanol. Therefore, a suitable organic solvent in preparation of TFC membrane through interfacial polymerization enables high pervaporation efficiency.

## 1. Introduction

Pervaporation, a membrane separation technique, consumes lesser energy than the traditional distillation process. Using pervaporation in purification of solvents or biofuels leads to a more affordable and greener approach. Hydrophilic membranes are engaged in the dehydration of solvents such as alcohols, acetic acid, and tetrahydrofuran [1]. Common hydrophilic polymers for pervaporation are chitosan [2,3,4], sodium alginate [5,6,7], and polyvinyl alcohol [8,9,10]. However, they are susceptible to swell in water that results in poor separation efficiency for a long period of time. Another hydrophilic material is polyamide. Polyamides are synthesized through polycondensation of amines with acyl chlorides [11]. In fabricating the polyamide membrane, two methods can be utilized. The first method is to synthesize the polyamide, then dissolve the polyamide in its solvent. The solvent used is dependent on the chemical structure and molecular weight of the polyamide. After dissolving the polyamide in the solvents, it can be cast in a plate to obtain a membrane. The second method is through the deposition of a polyamide layer on top of porous support by interfacial polymerization, dip coating or chemical cross-linking.

The most convenient approach is through interfacial polymerization between amine and acyl chloride on top of the porous support to create a thin and dense layer—usually called thin-film composite (TFC) membrane. Interfacial polymerization reaction occurred quickly to form a 5–500 nm thin layer on porous support [12]. The surface property of the layer formed is dependent on several factors: monomer concentration and structure [13,14,15], solvent property [16,17,18,19,20,21,22], fabrication method [23,24,25], membrane support property [26,27,28,29] and additives in the aqueous or organic phase [30,31,32,33]. The affinity of organic solvents with water and the amine monomer could influence the formation of the polyamide layer [19,20,21].

Different organic solvents have been explored in literature—toluene, xylene, hexane, heptane, cyclohexane, isopar G and isoparaffins [19,20,21]. Kim et al. [19] found that using isoparaffins produced a denser layer than using hexane. This was because when they dried the membrane at room temperature, hexane evaporates faster than isoparaffins, thus, the reaction could not be continued. Ghosh et al. [20] found that changing the organic solvent, which can give high diffusivity and solubility of m-phenylenediamine (MPD) to the organic solvents, the polyamide layer exhibited less cross-linked structure with high flux, membrane thickness and roughness but lower salt rejection. Park et al. [21] explored toluene, xylene, and hexane as organic solvents. They demonstrated that different organic solvents used for interfacial polymerization produce different surface morphology. MPD can diffuse faster with the reaction interface when using toluene or xylene than using hexane because toluene or xylene has higher affinity towards water. Other studies utilized cosolvent to decrease the immiscibility gap in the interface of water and organic solvent, which led to loose active layer [22]. However, most of the studies are for reverse osmosis membrane, thus, controlling the polyamide layer to a less cross-linked structure could increase the permeation flux.

In this study, four different organic solvents are considered in preparing a TFC pervaporation membrane. These are tetralin, toluene, hexane, and isopentane. Their affinity with water and DETA (diethylenetriamine) is different. DETA and TMC (trimesoyl chloride) undergo interfacial polymerization on top of the porous cellulose acetate (CA) support. The change in surface property and free volume of the membrane were investigated. Furthermore, these characteristics correlate with the membrane performance.

## 2. Materials and Methods

### 2.1. Materials

CA (394-60S) was received from Eastman (Palo Alto, CA, USA). N-Methyl-2-pyrrolidone (NMP), solvent of CA, was delivered by Tedia Company Inc., Fairfield, OH, USA. DETA and TMC were supplied by Tokyo Chemical Industry Co., Ltd. (Tokyo, Japan). Different solvents for TMC were tetralin (Sigma Aldrich, Saint Louis, MO, USA), toluene (Echo Chemical Co., Ltd., Taoyuan, Taiwan), hexane (Tedia Company Inc., Fairfield, OH, USA) and isopentane (Tedia Company Inc., Fairfield, OH, USA). Alcohols used for pervaporation such as methanol, ethanol, isopropanol, and tert-butanol, were all supplied by Echo Chemical Co., Ltd. (Taoyuan, Taiwan). Liquid nitrogen and helium were bought at Yang Special Gas Co., Ltd. (Taoyuan, Taiwan).

### 2.2. Fabrication of Thin-Film Composite Membrane

CA support was prepared through wet-phase inversion method. CA powder was dissolved in NMP solution (15 wt% CA in NMP) for 24 h. Afterwards, the solution was degassed overnight at room temperature. Then, the CA solution was cast onto a glass plate covered by polyester (PET) nonwoven using a 200 µm casting knife. The CA-coated PET nonwoven support was immediately immersed in a water coagulation bath to obtain the CA support. Ultimately, the CA supports were washed with water several times to remove the residual NMP.

**Figure 1:**
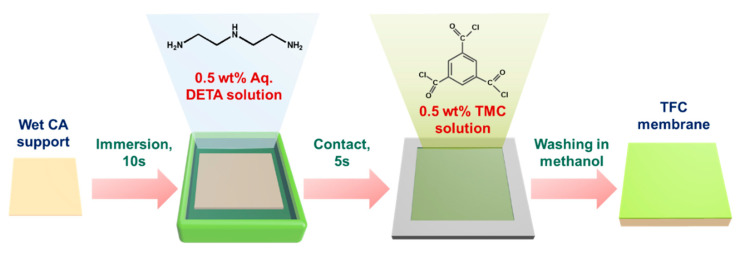
Schematic diagram for membrane preparation.

Prior to interfacial polymerization (Figure 1), the CA support was cut into 12 cm × 12 cm and was immersed in a 0.5 wt% aqueous DETA solution. After 10 s, the excess DETA solution on the surface of the CA support was removed using a rubber roller. The DETA-saturated CA support was clamped into an iron plate. This was followed by pouring a different organic solution containing 0.5 wt% TMC for 5 s. Afterwards, the membrane was soaked in methanol overnight to remove the unreacted monomers, then it was dried at room temperature. The membrane was designated as TFC_X_, where X refers to the type of organic solvent used (tetralin, toluene, hexane, or isopentane).

### 2.3. Characterization

Chemical analysis was performed using attenuated total reflectance-Fourier transform infrared (ATR-FTIR) spectroscopy (Perkin Elmer Spectrum 100 FTIR Spectrometer, Waltham, MA, USA) and X-ray photoelectron spectroscopy (XPS, VG K-alpha ThermoFisher Scientific, Inc., Waltham, MA, USA). Membrane morphology and surface roughness were observed using field emission scanning electron microscopy (FESEM, S-4800, Hitachi Co., Tokyo, Japan) and atomic force microscopy (AFM, NanoScope^®^ V, Bruker, Billerica, MA, USA), respectively. Hydrophilicity of the membrane was measured using an automatic interfacial tensiometer (PD-VP Model, Kyowa Interface Science Co., Ltd., Niiza City, Saitama, Japan). Free volume of the membrane was investigated through positron annihilation spectroscopy (PAS, R&D Center for Membrane Technology, Chung Yuan Christian University, Taoyuan, Taiwan).

### 2.4. Pervaporation Experiment

The pervaporation setup was similar to our previous work [34]. The sample was placed on the membrane cell with an effective surface area (*A*) of 11.64 cm^2^. The process was first stabilized for 1.5 h. Afterwards, permeate was collected from a small trap that was immersed in liquid nitrogen for 10 min. For each membrane condition, at least four pieces of membrane were fabricated to determine the membrane performance. The composition of permeate was determined using a gas chromatography analyzer (China Chromatography 9800, China Chromatography Co., Ltd., Taipei, Taiwan). The permeation flux (*J*) was calculated using Equation (1):

(1) $$J=\frac{W}{At}$$

where *W* was the weight of the collected permeate at time *t*. Separation factor (*β*) and pervaporation separation index (*PSI*) was determined using Equations (2) and (3), respectively.

(2) $$\beta =\frac{Y_{W}/Y_{A}}{X_{W}/X_{A}}$$

(3) PSI=J × β

where *Y*_w_ and *X*_w_ represented the respective concentration of water in permeate and feed; *Y*_A_ and *X*_A_, represented the respective concentration of alcohol in permeate and feed.

## 3. Results and Discussion

### 3.1. Surface Chemical Analysis

**Figure 2:**
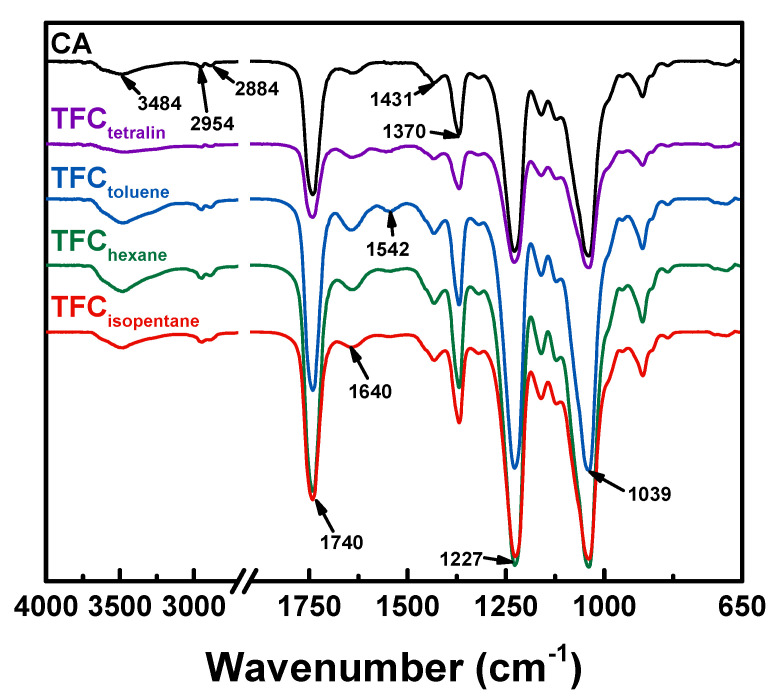
ATR-FTIR spectra of CA and TFC membranes.

Figure 2 indicates the ATR-FTIR spectra of CA and TFC membranes. CA had O–H stretching vibration at 3484 cm^−1^. C–H asymmetric and symmetric stretching vibration of CA were situated at 2954 and 2884 cm^−1^, respectively. The peaks at 1431 and 1370 cm^−1^ corresponded to symmetric and asymmetric bending of C–H, respectively. The C–O stretching of CA were located at 1227 cm^−1^, whereas the pyranose ring (C–O–C stretching) of CA were positioned at 1039 cm^−1^ [35]. After interfacial polymerization of DETA with TMC, a new peak was found at 1542 cm^−1^, corresponding to amide II (N–H). The amide I (C=O) of the TFC membranes overlapped with the spectra of CA at 1640 cm^−1^. Amide I and amide II both came from the cross-linking of DETA with TMC. However, when a different solvent of TMC was used, there was no significant change in the spectra of the TFC membranes. Therefore, the elemental surface composition (Table 1) of the membranes were examined using XPS analysis.

Table 1 summarizes the atomic composition of CA and TFC membranes. High N/O ratio means that the surface of TFC membranes comprises more cross-linked amide group or more unreacted amines of DETA. If the ratio of N/O is low, the surface of the polyamide would have more linear structure, which come from the hydrolysis of TMC. The N/O ratio of the TFC membranes follows this order: TFC_tetralin_ (0.4995) > TFC_toluene_ (0.3655) > TFC_hexane_ (0.2670) > TFC_isopentane_ (0.1026). These trends follow a similar trend with the viscosity of the organic solvents (Table 2), where tetralin is the most viscous and isopentane is the least viscous. If the TMC molecules were dissolved in more viscous organic solvents, during interfacial polymerization, the movement of TMC molecules to the immiscible interface is slow. These led to a polyamide layer with less cross-linked structure and more unreacted amines of DETA. On the other hand, when TMC was dissolved in less viscous solvent, it is easier to transport to the reaction interface, and could form denser or thicker polyamide. Hence, TFC_tetralin_ could have more unreacted amines and thin polyamide layer, whereas TFC_isopentane_ could have a more linear structure from the hydrolysis of TMC with thick polyamide layer.

Solubility parameter distance between molecules clarifies the intermolecular interaction of the solvents and monomers (Table 2). The shorter the solubility parameter distance, the molecules are more likely to interact with each other. The shorter the solubility parameter distance of DETA with the solvents, DETA is more likely to interact with that solvent. During interfacial polymerization, the diffusivity of DETA to the organic solvents from fastest to slowest follows accordingly: tetralin > toluene > hexane > isopentane. However, the viscosity of the solvents shows an opposite trend. When fabricating TFC_tetralin_, more DETA was presented on the reaction interface, but less TMC could react with DETA because of the slow movement of TMC in tetralin, which led to a loose polyamide structure. For TFC_isopentane_, enough DETA could exist on the reaction interface and more TMC presented in the interface because the movement of TMC molecules in isopentane was fast, this could result in a thick polyamide layer with more linear structure on the surface. During the growth of the polyamide layer, the densest part is located nearest the CA support. When the densest polyamide layer was formed, it blocked the DETA molecules to cross-link with TMC. Unreacted acyl chloride of TMC could not react with DETA, which led to hydrolysis to form carboxyl groups; therefore, more oxygen functional groups on the surface of the membrane with low N/O ratio.

### 3.2. Morphology, Surface Roughness and Hydrophilicity

**Figure 3:**
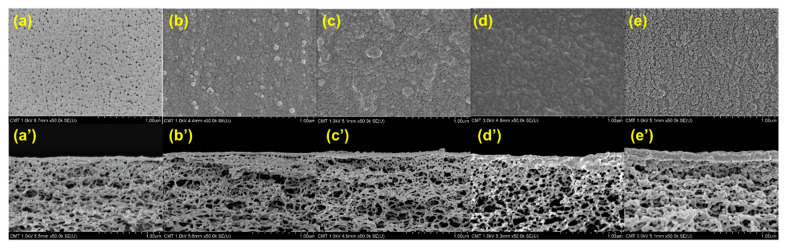
FESEM images of (**a**,**a’**) CA, (**b**,**b’**) TFC_tetralin_, (**c**,**c’**) TFC_toluene_, (**d**,**d’**) TFC_hexane_, and (**e**,**e’**) TFC_isopentane_.

Figure 3 presents the FESEM images of CA and TFC membranes. CA support had a very porous surface and substructure. After interfacial polymerization of DETA and TMC on its surface, pores were covered by the polyamide. Different organic solvents produced different surface morphology because of the difference in reaction rate of DETA and TMC. Protuberance or nodules were presented on all TFC membranes; however, they varied in shape and size. TFC_tetralin_, TFC_toluene_, and TFC_hexane_ had similar polyamide thickness, and they are thinner compared with TFC_isopentane._ As isopentane is less viscous, TMC diffused fast to the reaction interface, resulting in a thick polyamide layer.

**Figure 4:**
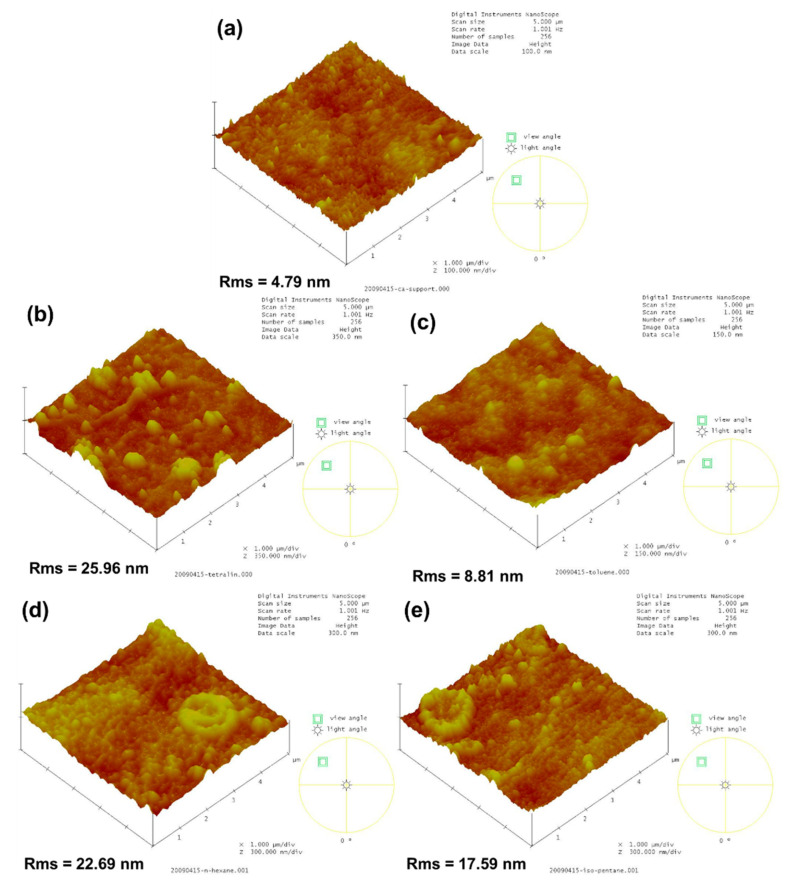
3-D AFM images of (**a**) CA, (**b**) TFC_tetralin_, (**c**) TFC_toluene_, (**d**) TFC_hexane_, and (**e**) TFC_isopentane_.

Figure 4 presents the 3-D AFM images of the membranes. The surface roughness of the membranes corresponded with the FESEM images, where TFC membranes had relatively rougher surface than that of CA support. Surface roughness and surface functional groups plays an important role in water contact angle. Table 3 lists the surface roughness and water contact angle. CA support had a hydrophilic surface with a contact angle of 45.40 ± 2.04°, because CA is abundant with hydroxyl groups. All TFC membranes had similar water contact angle range from 40.66 to 45.43°. There is no significant difference in their water contact angle because the surface roughness and surface property compensate each other. A hydrophilic membrane with rougher surface could provide a lower contact angle because rough surfaces have more surface area for the water to create contact [37]. Furthermore, TFC membranes are also abundant with several hydrophilic groups, such as amines, amides, and carboxyl groups. Therefore, the TFC membranes still had a hydrophilic property, which is favorable for dehydration of alcohols.

### 3.3. Free Volume Analysis Using Variable Monoenergy Slow Positron Beam

**Figure 5:**
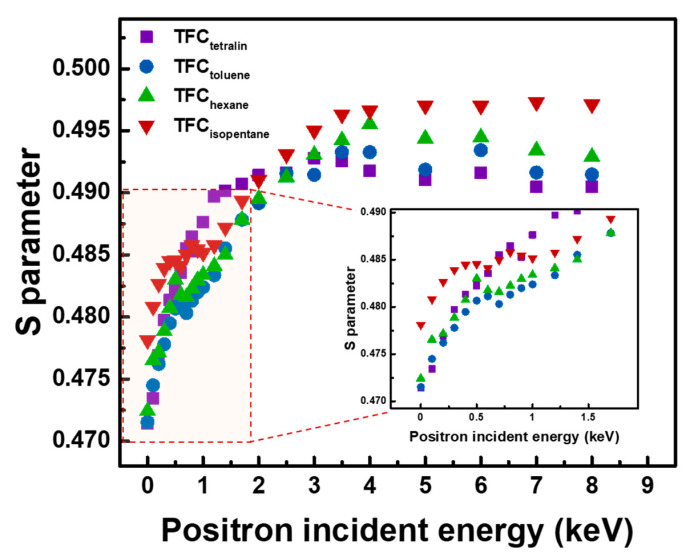
S parameter as a function of positron incident energy of TFC membranes.

Figure 5 shows the S parameter as a function of positron incident energy of the TFC membranes. From 0–0.5 keV, the steep rise of the S parameter came from the backscattering of the positron, which happens when the positrons create contact on the membrane surface. Comparing the TFC membranes, only TFC_isopentane_ had a plateau region from 0.5–1.5 keV, which represents the polyamide layer. Other membranes only had a plateau region from 0.5–0.7 keV, indicating that the polyamide layer formed on their surface is very thin. In general, higher S parameter could mean that the free volume is larger. TFC_isopentane_ not only had a higher S parameter than others, but also a thicker polyamide surface. The free volume and thickness of the polyamide layer could affect the membrane performance during pervaporation.

### 3.4. Performance of TFC Pervaporation Membranes

**Figure 6:**
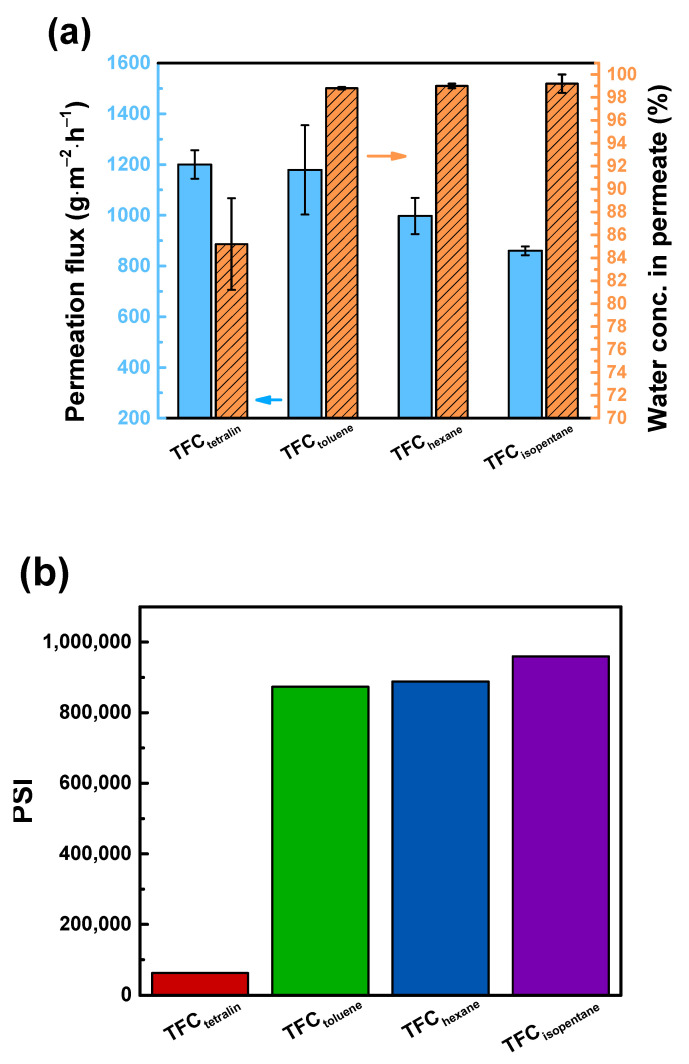
Effect of the organic solvents on (**a**) membrane performance and (**b**) pervaporation separation index. Feed = 90 wt% aqueous ethanol solution at 25 °C. Downstream pressure = 13 mmHg.

Figure 6 displays the performance of pervaporation membranes tested using 90 wt% aqueous ethanol solution. The permeation flux follows this order: TFC_tetralin_ (1200 ± 56 g∙m^−2^∙h^−1^) > TFC_toluene_ (1179 ± 176 g∙m^−2^∙h^−1^) > TFC_hexane_ (997 ± 71 g∙m^−2^∙h^−1^) > TFC_isopentane_ (860 ± 71 g∙m^−2^∙h^−1^). This trend was in similar order with the solubility parameter distance between solvent and water, and solvent and DETA. In addition, the concentration of water in permeate follows this decreasing order: TFC_isopentane_ (99.2 ± 0.8 wt%) > TFC_hexane_ (99.0 ± 0.2 wt%) > TFC_toluene_ (98.8 ± 0.1 wt%) > TFC_tetralin_ (85.2 ± 4.0 wt%). The permeation flux and separation efficiency show a trade-off phenomenon. Using organic solvents that have stronger affinity with the water and DETA, the membrane that was produced was looser, hence TFC_tetralin_ had the highest permeation flux but lowest separation efficiency. TFC_isopentane_ had the highest PSI value, because of its high separation efficiency with reasonable permeation flux. Furthermore, according to the analysis of PAS, TFC_isopentane_ had the thickest polyamide layer, thus, has the highest separation efficiency than other membranes. Therefore, the performance of TFC_isopentane_ membrane at different operating conditions is examined in the following section.

**Figure 7:**
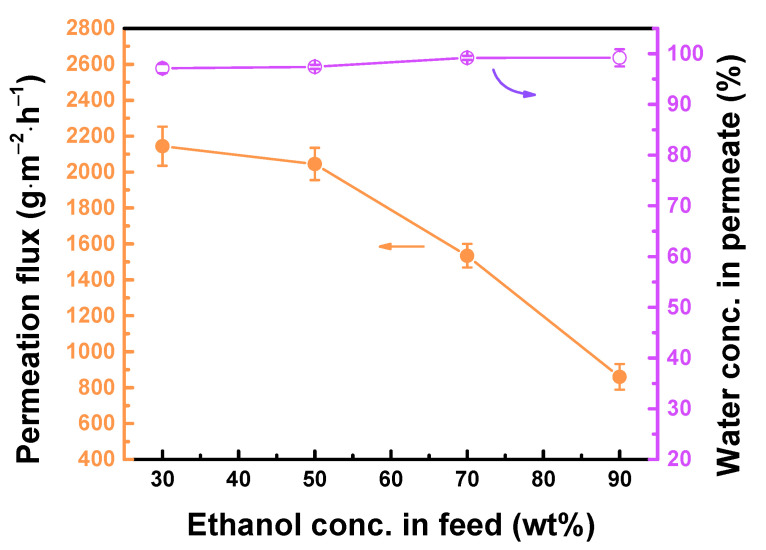
TFC_isopentane_ membrane performance at different feed concentration. Feed = 30−90 wt% aqueous ethanol solution at 25 °C. Downstream pressure = 13 mmHg.

Figure 7 plots the TFC_isopentane_ membrane performance at different concentrations of ethanol in the feed. Polyamide has a strong affinity with ethanol, hence, at a high concentration of ethanol, the ethanol could adsorb in the polymer, however, it is not easy to desorb. On the contrary, when the ethanol is adsorbed and becomes trapped into the wet zone of the polyamide, it would lead to a decrease in size of the pathway where the water molecules pass through the membrane, resulting in lower permeation flux. Therefore, with the ethanol concentration in the feed increased from 30 to 90 wt%, the permeation flux was decreased from 2144 ± 109 to 860 ± 71 g∙m^−2^∙h^−1^.

**Figure 8:**
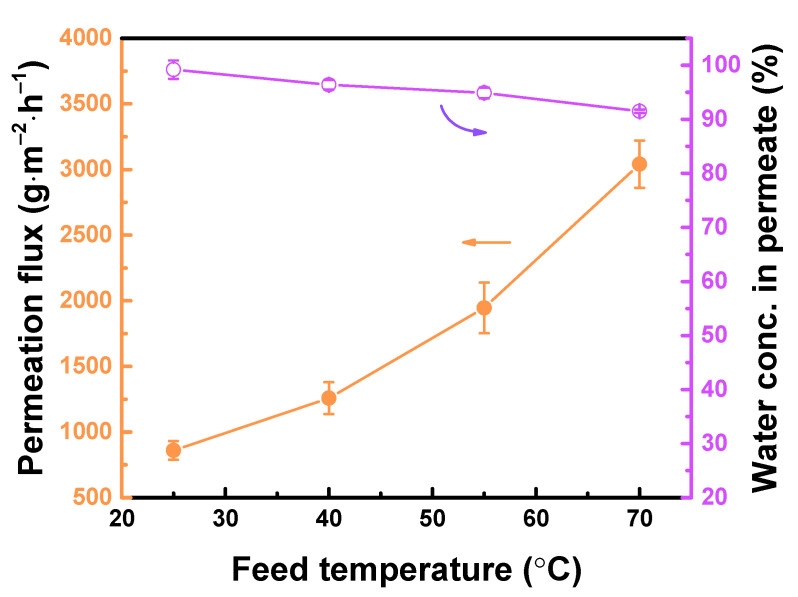
TFC_isopentane_ membrane performance at different operating feed temperature. Feed = 90 wt% aqueous ethanol solution. Downstream pressure = 13 mmHg.

Figure 8 demonstrates the effect of feed temperature on TFC_isopentane_ membrane performance. Increasing the feed temperature from 25 to 70 °C led to an increased in permeation flux from 860 ± 71 to 3041 ± 180 g∙m^−2^∙h^−1^. There is a larger driving force at high temperature, resulting in high permeation flux. However, there is also movement and enlargement of polymer chain, while the mobility of the water and alcohol was boosted, hence, more alcohol could penetrate through the membrane, resulting in water concentration in permeate decreased from 99.2 ± 1.7 to 91.5 ± 0.3 wt% [38].

**Figure 9:**
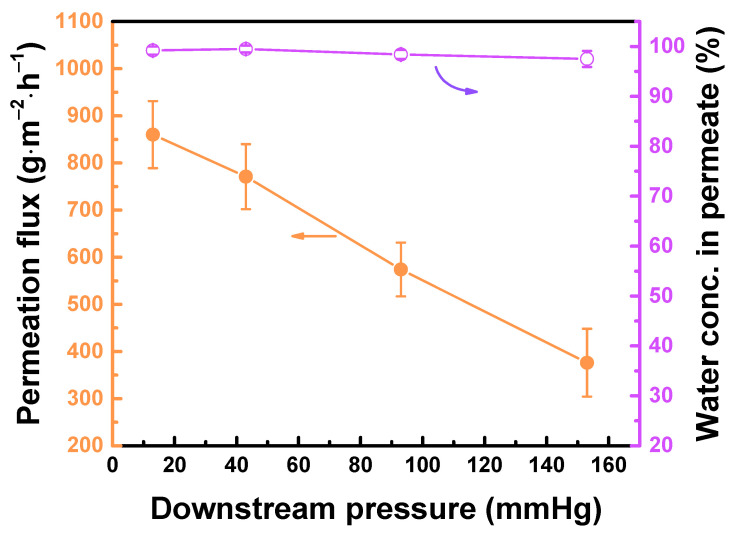
TFC_isopentane_ membrane performance at different downstream pressure. Feed = 90 wt% aqueous ethanol solution at 25 °C.

The downstream pressure affects the membrane performance because of the change in driving force (Figure 9). Increasing the downstream pressure from 13 to 153 mmHg led to a decrease in permeation flux from 860 ± 71 to 376 ± 72 g∙m^−2^∙h^−1^, but the water concentration in permeate remained high at 97–99 wt%. These results were attributed in the weakening of driving force at high downstream pressure. The desorption rate of molecules slowed down, resulting in low permeation flux.

**Figure 10:**
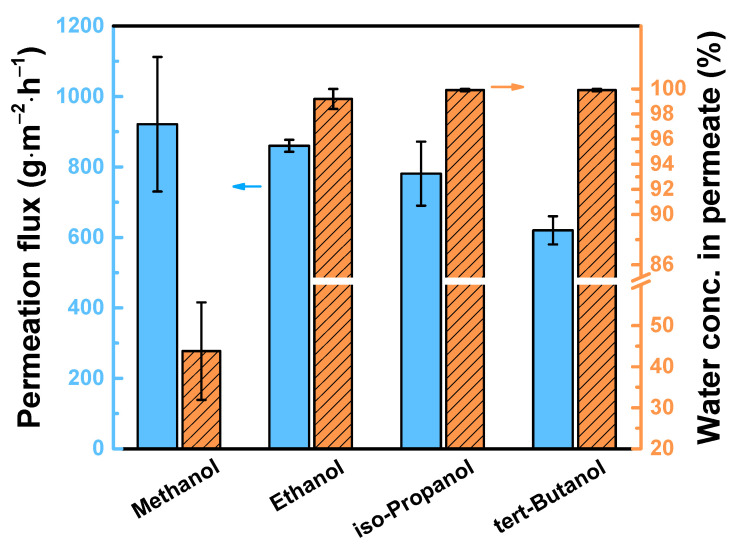
TFC_isopentane_ membrane performance at different feed alcohol solution. Feed = 90 wt% aqueous alcohol solution at 25 °C. Downstream pressure = 13 mmHg.

Figure 10 reveals the TFC_isopentane_ membrane performance at different feed alcohol. When the carbon number of the feed alcohol increased, the permeation flux of TFC_isopentane_ membrane decreased. This is because the molar volume of alcohol also increased, hence, it is not easy for the large volume of alcohol to pass through the membrane to permeate, resulting in lower permeation flux. The following are the molar volume of the alcohols: methanol (40.7 mL/mol) < ethanol (58.5 mL/mol) < isopropanol (76.5 mL/mol) < tert-butanol (92.4 mL/mol). When the feed is methanol, the concentration of water in permeate was only 43.3 ± 11.9 wt%, because methanol has a smaller molar volume, hence, it penetrated easily through the membrane. Moreover, when the feed was ethanol, isopropanol or tert-butanol, the concentration of water in permeate was 99.2 ± 0.8, 99.9 ± 0.1, and 99.9 ± 0.1 wt%, respectively. Table 4 compares the membrane performance from other literature and our work. Our membranes show a comparable performance with high permeation flux and selectivity. Therefore, this shows that TFC_isopentane_ membrane is promising for purification of bioalcohol.

## 4. Conclusions

TFC membranes were prepared through interfacial polymerization of DETA and TMC. The solvent for TMC was varied: tetralin, toluene, hexane, and isopentane. The affinity of organic solvents with water and DETA affects the physicochemical property and performance of TFC membranes. The ratio of N/O was dependent on the reaction rate of the monomers. The membrane prepared using isopentane produced the thickest polyamide layer, because isopentane has lower viscosity than the other solvents, which led to the favorable diffusion of TMC to the reaction interface. Even if DETA has a weak affinity with isopentane, the amount of DETA on the reaction interface was enough to produce a defect-free polyamide membrane for pervaporation of alcohols. Accordingly, TFC_isopentane_ had the highest separation efficiency in isopropanol dehydration with a stable performance at different operating conditions (feed alcohol, feed temperature, and downstream pressure). Furthermore, it could also be used for dehydrating ethanol and tert-butanol.

## Figures and Tables

**Table 1:** Atomic composition and N/O ratio of the membranes from XPS analysis.

Membrane	C (%)	O (%)	N (%)	N/O
CA	54.93	45.07	−	−
TFC_tetralin_	68.42	21.06	10.52	0.4995
TFC_toluene_	67.50	23.80	8.70	0.3655
TFC_hexane_	59.20	32.21	8.60	0.2670
TFC_isopentane_	55.19	40.65	4.17	0.1026

**Table 2:** Viscosity and Hansen solubility parameters of the compounds.

Compound	Viscosity (cp)	δ_d_ (MPa^1/2^)	δ_p_ (MPa^1/2^)	δ_h_ (MPa^1/2^)	δ_t_ (MPa^1/2^)	Ra_solvent-water_^c^	Ra_solvent-DETA_^c^
Water	0.895 ^a^	15.50	16.00	42.30	47.81	−	−
Tetralin	2.023 ^a^	19.60	2.00	2.90	19.91	42.61	17.07
Toluene	0.560 ^a^	18.00	1.40	2.00	18.16	43.15	17.31
Hexane	0.326 ^b^	14.90	0.00	0.00	14.90	45.24	19.86
Isopentane	0.214 ^b^	13.70	0.00	0.00	13.70	45.37	20.43
DETA	7.14 ^b^	16.70	13.30	14.30	25.70	−	−

^a^ Viscosity at 25 °C. ^b^ Viscosity at 20 °C. ^c^ Ra: solubility parameter distance. Solubility parameter reference [36].

**Table 3:** Water contact angle of the membranes.

Membrane	Contact Angle (°) ^a^
CA	45.40 ± 2.04
TFC_tetralin_	45.43 ± 1.58
TFC_toluene_	39.46 ± 1.75
TFC_n-hexane_	42.09 ± 1.51
TFC_iso-pentane_	40.66 ± 2.36

^a^ Measured after 1 min.

**Table 4:** A comparison of membrane performance in our work with other reported literature.

Membrane	Feed Ethanol Conc. (wt%)	Operating Temperature (°C)	Permeation Flux (g∙m^−2^∙h^−1^)	Water Conc. in Permeate (wt%)	Ref.
DETA/TMC	90	25	860	99.2	This study
DAPL-SCC/mPAN	90	25	600	96.7	[13]
m-tolidine-H-TMC/mPAN TFC	90	25	2191	99.5	[39]
PAA-PA/PAN	90	25	830	99.5	[40]
PA+nano-NaX zeolite/mPAN	90	25	4500	77	[41]
SA/PFSA/ceramic hybrid membrane	85	75	821	99.9	[42]
TDI cross-linked PA	85	50	2000	95.9	[43]
PVA-P4-80 hybrid	85	40	145	99.5	[44]

## References

[1] Wang L., Wang Y., Wu L., Wei G. (2020). Fabrication, properties, performances, and separation application of polymeric pervaporation membranes: A review. Polymers.

[2] Xu Z., Liu G., Ye H., Jin W., Cui Z. (2018). Two-dimensional mxene incorporated chitosan mixed-matrix membranes for efficient solvent dehydration. J. Membr. Sci..

[3] Castro-Muñoz R., González-Valdez J., Ahmad M.Z. (2020). High-performance pervaporation chitosan-based membranes: New insights and perspectives. Rev. Chem. Eng..

[4] Du J.R., Hsu L.H., Xiao E.S., Guo X., Zhang Y., Feng X. (2020). Using genipin as a “green” crosslinker to fabricate chitosan membranes for pervaporative dehydration of isopropanol. Sep. Purif. Technol..

[5] Veerapur R.S., Gudasi K.B., Patil M.B., Babu V.R., Bhat S.D., Sairam M., Aminabhavi T.M. (2006). Sodium alginate–poly(hydroxyethylmethacrylate) interpenetrating polymeric network membranes for the pervaporation dehydration of ethanol and tetrahydrofuran. J. Appl. Polym. Sci..

[6] Mokhtarzadeh S., Hakimpour F., Sarvari R., Agbolaghi S., Mansourpanah Y. (2020). Nanocomposite membranes based on sodium alginate/poly(ε-caprolactone)/graphene oxide for methanol, ethanol and isopropanol dehydration via pervaporation. Polym. Bull..

[7] Zhao J., Zhu Y., He G., Xing R., Pan F., Jiang Z., Zhang P., Cao X., Wang B. (2016). Incorporating zwitterionic graphene oxides into sodium alginate membrane for efficient water/alcohol separation. ACS Appl. Mater. Interfaces.

[8] Chaudhari S., Kwon Y.S., Shon M.Y., Nam S.E., Park Y. (2020). Surface-modified polyvinyl alcohol (PVA) membranes for pervaporation dehydration of epichlorohydrin (ECH), isopropanol (IPA), and water ternary feed mixtures. J. Ind. Eng. Chem..

[9] Kwon Y.S., Chaudhari S., Kim C.E., Son D.H., Park J.H., Moon M.J., Shon M.Y., Park Y., Nam S.E. (2018). Ag-exchanged nay zeolite introduced polyvinyl alcohol/polyacrylic acid mixed matrix membrane for pervaporation separation of water/isopropanol mixture. RSC Adv..

[10] Sajjan A.M., Jeevan Kumar B.K., Kittur A.A., Kariduraganavar M.Y. (2013). Development of novel grafted hybrid pva membranes using glycidyltrimethylammonium chloride for pervaporation separation of water–isopropanol mixtures. J. Ind. Eng. Chem..

[11] Morgan P.W. (1965). Condensation Polymers: By Interfacial and Solution Methods.

[12] Raaijmakers M.J., Benes N.E. (2016). Current trends in interfacial polymerization chemistry. Prog. Polym. Sci..

[13] An Q.F., Ang M.B.M.Y., Huang Y.H., Huang S.H., Chia Y.H., Lai C.L., Tsai H.A., Hun W.S., Hu C.C., Wu Y.P. (2019). Microstructural characterization and evaluation of pervaporation performance of thin-film composite membranes fabricated through interfacial polymerization on hydrolyzed polyacrylonitrile substrate. J. Membr. Sci..

[14] Liu Y.L., Zhao Y.Y., Wang X.M., Wen X.H., Huang X., Xie Y.F. (2019). Effect of varying piperazine concentration and post-modification on prepared nanofiltration membranes in selectively rejecting organic micropollutants and salts. J. Membr. Sci..

[15] Chiao Y.H., Sengupta A., Chen S.T., Huang S.H., Hu C.C., Hung W.S., Chang Y., Qian X., Wickramasinghe S.R., Lee K.R. (2019). Zwitterion augmented polyamide membrane for improved forward osmosis performance with significant antifouling characteristics. Sep. Purif. Technol..

[16] Marquez J.A.D., Ang M.B.M.Y., Doma B.T., Huang S.H., Tsai H.A., Lee K.R., Lai J.Y. (2018). Application of cosolvent-assisted interfacial polymerization technique to fabricate thin-film composite polyamide pervaporation membranes with pvdf hollow fiber as support. J. Membr. Sci..

[17] Lee J., Wang R., Bae T.H. (2019). A comprehensive understanding of co-solvent effects on interfacial polymerization: Interaction with trimesoyl chloride. J. Membr. Sci..

[18] Yan W., Wang Z., Zhao S., Wang J., Zhang P., Cao X. (2019). Combining co-solvent-optimized interfacial polymerization and protective coating-controlled chlorination for highly permeable reverse osmosis membranes with high rejection. J. Membr. Sci..

[19] Kim I.C., Jegal J., Lee K.H. (2002). Effect of aqueous and organic solutions on the performance of polyamide thin-film-composite nanofiltration membranes. J. Polym. Sci. Pt. B-Polym. Phys..

[20] Ghosh A.K., Jeong B.H., Huang X., Hoek E.M.V. (2008). Impacts of reaction and curing conditions on polyamide composite reverse osmosis membrane properties. J. Membr. Sci..

[21] Park S.J., Kwon S.J., Kwon H.E., Shin M.G., Park S.H., Park H., Park Y.I., Nam S.E., Lee J.H. (2018). Aromatic solvent-assisted interfacial polymerization to prepare high performance thin film composite reverse osmosis membranes based on hydrophilic supports. Polymer.

[22] Esfandian F., Peyravi M., Ghoreyshi A.A., Jahanshahi M., Rad A.S. (2019). Fabrication of tfc nanofiltration membranes via co-solvent assisted interfacial polymerization for lactose recovery. Arab. J. Chem..

[23] Huang S.H., Hung W.S., Liaw D.J., Tsai H.A., Jiang G.J., Lee K.R., Lai J.Y. (2010). Positron annihilation study on thin-film composite pervaporation membranes: Correlation between polyamide fine structure and different interfacial polymerization conditions. Polymer.

[24] Tsai H.A., Chen Y.L., Huang S.H., Hu C.C., Hung W.S., Lee K.R., Lai J.Y. (2018). Preparation of polyamide/polyacrylonitrile composite hollow fiber membrane by synchronous procedure of spinning and interfacial polymerization. J. Membr. Sci..

[25] Chiao Y.H., Chen S.T., Patra T., Hsu C.H., Sengupta A., Hung W.S., Huang S.H., Qian X., Wickramasinghe R., Chang Y. (2019). Zwitterionic forward osmosis membrane modified by fast second interfacial polymerization with enhanced antifouling and antimicrobial properties for produced water pretreatment. Desalination.

[26] Ang M.B.M.Y., Lau V., Ji Y.L., Huang S.H., An Q.F., Caparanga A.R., Tsai H.A., Hung W.S., Hu C.C., Lee K.R. (2017). Correlating PSf support physicochemical properties with the formation of piperazine-based polyamide and evaluating the resultant nanofiltration membrane performance. Polymers.

[27] Huang L., McCutcheon J.R. (2015). Impact of support layer pore size on performance of thin film composite membranes for forward osmosis. J. Membr. Sci..

[28] Yakavalangi M.E., Rimaz S., Vatanpour V. (2017). Effect of surface properties of polysulfone support on the performance of thin film composite polyamide reverse osmosis membranes. J. Appl. Polym. Sci..

[29] Ghosh A.K., Hoek E.M.V. (2009). Impacts of support membrane structure and chemistry on polyamide–polysulfone interfacial composite membranes. J. Membr. Sci..

[30] Ang M.B.M.Y., Ji Y.L., Huang S.H., Lee K.R., Lai J.Y. (2019). A facile and versatile strategy for fabricating thin-film nanocomposite membranes with polydopamine-piperazine nanoparticles generated in situ. J. Membr. Sci..

[31] Ang M.B.M.Y., Tang C.L., De Guzman M.R., Maganto H.L.C., Caparanga A.R., Huang S.H., Tsai H.A., Hu C.C., Lee K.R., Lai J.Y. (2020). Improved performance of thin-film nanofiltration membranes fabricated with the intervention of surfactants having different structures for water treatment. Desalination.

[32] Kim I.C., Jeong B.R., Kim S.J., Lee K.H. (2013). Preparation of high flux thin film composite polyamide membrane: The effect of alkyl phosphate additives during interfacial polymerization. Desalination.

[33] Yung L., Ma H., Wang X., Yoon K., Wang R., Hsiao B.S., Chu B. (2010). Fabrication of thin-film nanofibrous composite membranes by interfacial polymerization using ionic liquids as additives. J. Membr. Sci..

[34] Ang M.B.M.Y., Huang S.H., Li Y.C., Cahatol A.T.C., Tayo L.L., Hung W.S., Tsai H.A., Hu C.C., Lee K.R., Lai J.Y. (2020). High-performance thin-film composite polyetheramide membranes for the dehydration of tetrahydrofuran. J. Membr. Sci..

[35] Lv J., Zhang G., Zhang H., Yang F. (2017). Exploration of permeability and antifouling performance on modified cellulose acetate ultrafiltration membrane with cellulose nanocrystals. Carbohydr. Polym..

[36] Hansen C.M. (2007). Hansen Solubility Parameters: A User’s Handbook.

[37] Wenzel R.N. (1936). Resistance of solid surfaces to wetting by water. Ind. Eng. Chem..

[38] Huang C.H., Liu Y.L. (2017). Self-healing polymeric materials for membrane separation: An example of a polybenzimidazole-based membrane for pervaporation dehydration on isopropanol aqueous solution. RSC Adv..

[39] Huang Y.H., Huang S.H., Chao W.C., Li C.L., Hsieh Y.Y., Hung W.-S., Liaw D.J., Hu C.C., Lee K.R., Lai J.Y. (2014). A study on the characteristics and pervaporation performance of polyamide thin-film composite membranes with modified polyacrylonitrile as substrate for bioethanol dehydration. Polym. Int..

[40] Ang M.B.M.Y., Huang S.H., Chang M.W., Lai C.L., Tsai H.A., Hung W.S., Hu C.C., Lee K.R. (2020). Ultraviolet-initiated graft polymerization of acrylic acid onto thin-film polyamide surface for improved ethanol dehydration performance of pervaporation membranes. Sep. Purif. Technol..

[41] Fathizadeh M., Aroujalian A., Raisi A., Fotouhi M. (2013). Preparation and characterization of thin film nanocomposite membrane for pervaporative dehydration of aqueous alcohol solutions. Desalination.

[42] Xie H.R., Ji C.H., Xue S.M., Xu Z.L., Yang H., Ma X.H. (2018). Enhanced pervaporation performance of sa-pfsa/ceramic hybrid membranes for ethanol dehydration. Sep. Purif. Technol..

[43] Zuo J., Lai J.Y., Chung T.S. (2014). In-situ synthesis and cross-linking of polyamide thin film composite (tfc) membranes for bioethanol applications. J. Membr. Sci..

[44] Xia L.L., Li C.L., Wang Y. (2016). In-situ crosslinked PVA/organosilica hybrid membranes for pervaporation separations. J. Membr. Sci..

